# Estabilidad del ph de cuatro geles a base de peróxido de hidrógeno en distintos intervalos de tiempo

**DOI:** 10.21142/2523-2754-0902-2021-058

**Published:** 2021-06-21

**Authors:** Claudia Andrea Ruiz Gamero

**Affiliations:** 1 Facultad de Estomatología, Universidad Inca Garcilaso de la Vega. Lima, Perú. carg1003@hotmail.com Universidad Inca Garcilaso de la Vega Facultad de Estomatología Universidad Inca Garcilaso de la Vega Lima Peru carg1003@hotmail.com; 2 Especialidad de Odontología Estética y Restauradora, Universidad Científica del Sur. Lima, Perú. Universidad Científica del Sur Especialidad de Odontología Estética y Restauradora Universidad Científica del Sur Lima Peru

**Keywords:** aclaramiento dental, pH, peróxido de hidrógeno, bleaching agent, pH, hydrogen peroxide

## Abstract

**Objetivo:**

Comparar el pH de cuatro marcas de geles aclaradores a base de peróxido de hidrógeno de altas concentraciones (30%-35%) Whiteness HP Maxx (HPM), Lase Peroxide (LP), Whiteness HP Automixx (HPA)y Dash (DA), a través del tiempo de aplicación clínica (inicio, 15’, 30’ y 45’).

**Materiales y métodos:**

El estudio fue experimental *in vitro*. Se evaluaron 40 muestras (dientes bovinos) divididos en 4 grupos, uno para cada marca comercial de gel. Se preparó cada gel de acuerdo con las instrucciones del fabricante y se colocó una cantidad necesaria en la superficie vestibular; posteriormente, se registró el pH del gel con un pHmetro digital al inicio, 15, 30 y 45 minutos. Los datos se analizaron con las pruebas Anova, Friedman y Wilcoxon.

**Resultados:**

Hubo una tendencia a la disminución del pH desde el tiempo inicial de aplicación hasta el tiempo final, con excepción del grupo de la marca DA, el cual mostró que los valores del pH fueron aumentando a través del tiempo. En la marca HPM, existe una diferencia significativa entre el tiempo inicial de aplicación del gel y el resto de tiempos. En la marca LP, a partir de los 15’ de aplicación, sí hay diferencia significativa entre los tiempos. En cuanto a la marca HPA, existieron diferencias significativas entre el tiempo inicial de aplicación y los demás tiempos. Finalmente, con la marca DA se encontró únicamente una diferencia significativa entre el tiempo inicial de aplicación y el tiempo final.

**Conclusiones:**

El pH de los geles aclaradores disminuyó a través del tiempo de aplicación clínica en todas las marcas estudiadas, con excepción de la marca Dash 30%, la cual mostró un aumento.

## Abstract (en)

**Objective:**

The aim of this *in vitro* study was to evaluate the pH of four bleaching agents based on high concentration hydrogen peroxide (30-35%) Whiteness HP Maxx (HPM), Lase Peroxide (LP), Whiteness HP Automixx (HPA) and Dash (DA) in different clinical periods (baseline, 15', 30' and 45').

**Materials and methods:**

40 specimens (bovine teeth) were divided into 4 groups; one group for each bleaching agent. Each bleaching agent was prepared according to the manufacturer's instructions and was applied on the vestibular surface. The pH of the bleaching agent was measured with a digital pH meter at baseline, 15, 30 and 45 minutes. ANOVA, Friedman and Wilcoxon tests were applied.

**Results:**

The pH values showed a trend to decreasing from the initial time of application to the final time, except for the DA group, which showed increasing pH values over time. The HPM group showed significant differences between baseline and the remaining periods. The LP group LP showed significant difference between 15' and the other periods. The HPA group showed significant differences between baseline and the remaining periods. Finally, the DA group, showed a significant difference between baseline and 45’.

**Conclusions:**

The pH values of 3 of the bleaching agents decreased over time, with the exception of Dash which increased in the different time periods.

## INTRODUCCIÓN

El aclaramiento dental es un método efectivo y conservador que ha sido usado desde hace muchos años. Tiene como objetivo aclarar los dientes que presentan alteraciones de color ^1^^,^^2^. El color dentario depende de la translucidez del esmalte y la dentina, así como de la reflexión de los colores en el diente ^3^. Se forma por compuestos orgánicos que poseen cadenas de enlaces simples y dobles, que forman un cromósfero ^4^^,^^5^. El aclaramiento actúa rompiendo estos enlaces para modificar el color.

De acuerdo con la percepción individual de cada paciente, estos colores pueden no ser estéticos, por lo que buscarán mejorarlos. A través del tiempo, desde la primera vez que empezaron a desarrollarse los diferentes métodos de aclaramiento dental, se investigaron y probaron distintos materiales y técnicas con el fin de protocolizar dicho tratamiento de una manera efectiva, segura y predecible ^6^.

Antes de realizar un aclaramiento dental, se debe evaluar la condición del paciente para determinar la técnica y el material a emplear. Se debe observar lo siguiente: presencia de caries dental (complejidad y ubicación); presencia de dientes oscurecidos; fisuras o cracs dentarios; descalcificaciones localizadas o manchas blancas; áreas translúcidas en bordes incisales que con el aclaramiento podrían intensificarse y crear un aspecto grisáceo, lo que afectaría el resultado final; restauraciones anteriores (se debe determinar si necesitarán retratamiento una vez finalizado el aclaramiento); simetría gingival (para optimizar el resultado final) y, por último, presencia de abrasiones, atriciones y recesiones, ya que éstas no cambiarán de color.

En cuanto al mecanismo de acción del aclaramiento, existe una teoría cromósfera ^1^ que se basa en la interacción del peróxido de hidrógeno con cromósferos orgánicos de la estructura dentaria. Estos cromósferos tienen áreas ricas en electrones y sus cadenas, al reaccionar, se convierten en estructuras más simples y alteran sus propiedades ópticas, lo cual disminuye la intensidad de la pigmentación ^3^^,^^5^^,^^7^.

En este proceso se pueden distinguir 3 fases: (1) el comportamiento del agente aclarador aplicado desde la superficie externa en el esmalte; (2) la interacción de las moléculas de pigmentos con el peróxido de hidrógeno tras su penetración a la estructura dental; y (3) los cambios micromorfológicos inducidos por el peróxido en la estructura dentaria, que finalmente producen cambios ópticos. Estas tres fases dan como resultado el cambio final del color del diente después del aclaramiento.

Los efectos de los agentes aclaradores en el esmalte han sido ampliamente estudiados. Entre ellos se encuentran los cambios de la morfología, el aumento de la porosidad superficial, la exposición de prismas, la reducción del contenido orgánico, los cambios en la proporción calcio/fosfato y la disminución de la microdureza. Todos dependerán del tiempo de permanencia del gel en el sustrato, la concentración del peróxido y el pH durante su uso ^5^^,^^8^^,^^11^.

El pH de los geles aclaradores de alta concentración de H2O2 oscila entre 3 y 7 ^14^, lo cual indica que hay productos que tienen un pH crítico para el esmalte (menor a 5,5). El pH de un producto aclarador tiene una relación directa con la rugosidad del esmalte una vez realizado el aclaramiento, y tiende a disminuir conforme más tiempo pase en contacto con el sustrato dentario ^8^^-^^9^^,^^13^^-^^15^.

Actualmente existen muchos productos de aclaramiento dental a base de peróxido de hidrógeno, que se diferencian entre sí por su concentración (la cual puede estar entre el 10% y el 40%) y su pH. Asimismo, algunos geles contienen otros componentes, como gluconato de calcio, fosfatos cálcicos y agentes desensibilizantes. Estos sistemas también varían en su modo de aplicación: en algunos se debe cambiar el producto cada 15 minutos y otros pueden permanecer por 40-50 minutos sin cambiarse. 

Es importante que los productos aclaradores tengan un pH relativamente alcalino para minimizar los riesgos potenciales. La mayoría presenta pH ácido debido a que el H2O2 se conserva mejor en este medio, por lo que al fabricar los geles se les adiciona un ácido débil para evitar su descomposición.

Diversos estudios reportan que el gel aclarador a base de peróxido de hidrógeno que tenga un pH básico será más efectivo, ya que la disociación constante del H2O2 será mayor que en una solución ácida ^1^. También han observado que la eficacia del H2O2 es directamente proporcional al incremento del pH del gel aclarador; aunque clínicamente no es significativo en cuanto a color, sí lo es en cuanto a sensibilidad. Adicionalmente, también es importante mencionar que los geles aclaradores alcalinos usualmente muestran un pH más estable a través del tiempo que los ácidos ^1^.

El criterio del odontólogo para elegir un gel aclarador y su concentración se basa en los resultados que busca sin alterar o afectar el tejido dentario, y el tiempo que pretende invertir. Resulta de vital importancia para el odontólogo conocer a fondo las propiedades de las distintas opciones que existen actualmente, como las diferentes marcas de geles aclaradores a base peróxido de hidrógeno. Se deben conocer dichas propiedades para saber cómo se van a comportar a través del tiempo de aplicación clínica; de esta manera, se podrá establecer un protocolo de uso. Es importante establecer uno que vaya más allá de las instrucciones del fabricante (aunque sin dejar de respetarlas) y que esté de acuerdo con el tipo de peróxido y su concentración y variación de pH. De esta manera se podrán optimizar las propiedades de cada peróxido y lograr el éxito en el tratamiento aclarador.

El objetivo de esta investigación fue comparar el pH de 4 marcas de geles aclaradores a base de peróxido de hidrógeno de alta concentración (30%-35%): Whiteness HP Maxx (HPM), Lase Peroxide (LP), Whiteness HP Automixx (HPA)y Dash (DA), a través del tiempo de aplicación clínica (inicio, 15’, 30’ y 45’).

## MATERIALES Y MÉTODOS

El estudio fue de tipo experimental *in vitro*. La muestra estuvo conformada por 48 dientes bovinos y 4 geles aclaradores a base de peróxido de hidrógeno, que cumplieron con los criterios de inclusión de la investigación: dientes bovinos sanos y geles aclaradores a base de peróxido de hidrógeno con una concentración igual o mayor al 30%. 

La investigadora principal se capacitó en la correcta manipulación de los geles aclaradores a base de peróxido de hidrógeno de altas concentraciones con el Dr. Gustavo Watanabe. Además, la investigadora fue capacitada por personal de la empresa Bionet S. A. en la manipulación adecuada del pHmetro utilizado (Pasco Extech).

Fueron utilizados 4 geles aclaradores a base de peróxido de hidrógeno con una concentración del 30% a más: Whiteness HP Maxx 35% de FGM lote 080916, con fecha de vencimiento setiembre de 2018; Lase Peroxide Sensy 35% de DMC Lote 21754, con fecha de vencimiento setiembre de 2018; Whiteness HP Automixx 35% de FGM Lote 271018, con fecha de vencimiento octubre de 2018, y Dash 30% de Philips Lote DSE1001, con fecha de vencimiento diciembre de 2017.

Los 48 dientes bovinos fueron distribuidos aleatoriamente en 4 grupos, cada uno correspondiente a una marca de gel aclarador. 

Para la medición del pH se utilizó un pHmetro digital marca Pasco Extech, el cual registra los valores de pH por segundo de contacto del electrodo del aparato con el gel de cada muestra. El pHmetro fue calibrado con soluciones básicas antes de cada experimento. Por otro lado, el electrodo fue lavado con agua destilada y secado con papel tisú entre muestra y muestra, con el fin de prevenir cualquier contaminación entre prueba y prueba. Una vez colocado el gel aclarador en cada muestra por grupo, se procedió a contactar cada una con el electrodo del pHmetro durante 5 segundos, con el fin de que este registre los valores y así poder trasladarlos a la ficha de recolección de datos en los 4 intervalos de tiempo (0’, 15’, 30’ y 45’).

El análisis estadístico de los datos se realizó utilizando el *software* IBM SPSS versión 23.0. Se emplearon las pruebas no paramétricas de Friedman y Wilcoxon. Solo el grupo 4 (Dash) tuvo distribución normal, por lo que se utilizó la prueba de Anova.

Se estableció que el valor “P”, para determinar la diferencia estadísticamente significativa, sería menor a 0,05.

## RESULTADOS

En el análisis descriptivo de la variable pH, se observó que este fue descendiendo a través de los cuatro intervalos de tiempo de aplicación clínica en 3 marcas de los geles aclaradores: Whiteness HP 35% (Tabla 1), Lase Peroxide Sensy 35% (Tabla 2) y Whiteness HP Automixx 35% (Tabla 3); sin embargo, el pH del gel clareador Dash (Tabla 4) mostró un incremento a través del tiempo de aplicación clínica. 

**Tabla 1:** Evaluación descriptiva del pH del gel aclarador Whiteness HP Automixx 35%, Lase Peroxide 35%, Whiteness HP Automixx 35% y Dash 30% al momento inicial (0’) de aplicación clínica

GEL / TIEMPO	0'					
	N	Media	DE	S^2^	Mín	Máx
Whiteness HP Maxx 35%	12	5,99	0,133	0,018	5,84	6,27
Lase Peroxide 35%	12	6,34	0,400	0,160	5,24	6,73
Whiteness HP Automixx 35%	12	6,65	0,493	0,243	5,96	7,68
Dash 30%	12	5,71	0,404	0,163	5,20	6,55

**Tabla 2:** Evaluación descriptiva del pH del gel aclarador Whiteness HP Automixx 35%, Lase Peroxide 35%, Whiteness HP Automixx 35% y Dash 30% a los 15’ de aplicación clínica

GEL / TIEMPO	15'					
	N	Media	DE	S^2^	Mín	Máx
Whiteness HP Maxx 35%	12	5,66	0,153	0,024	5,50	6,1
Lase Peroxide 35%	12	6,00	0,137	0,019	5,79	6,35
Whiteness HP Automixx 35%	12	6,00	0,327	0,108	5,79	6,97
Dash 30%	12	6,00	0,532	0,284	5,19	6,91

**Tabla 3:** Evaluación descriptiva del pH del gel aclarador Whiteness HP Automixx 35%, Lase Peroxide 35%, Whiteness HP Automixx 35% y Dash 30% a los 30’ de aplicación clínica

GEL / TIEMPO	30'					
	N	Media	DE	S^2^	Mín	Máx
Whiteness HP Maxx 35%	12	5,55	0,074	0,005	5,47	5,67
Lase Peroxide 35%	12	5,74	0,081	0,007	5,62	5,93
Whiteness HP Automixx 35%	12	5,67	0,198	0,039	5,34	6,18
Dash 30%	12	6,07	0,366	0,134	5,64	6,79

**Tabla 4:** Evaluación descriptiva del pH del gel aclarador Whiteness HP Automixx 35%, Lase Peroxide 35%, Whiteness HP Automixx 35% y Dash 30% a los 45’ de aplicación clínica

GEL / TIEMPO	45'					
	N	Media	DE	S^2^	Mín	Máx
Whiteness HP Maxx 35%	12	5,46	0,089	0,008	5,30	5,61
Lase Peroxide 35%	12	5,62	0,078	0,006	5,54	5,79
Whiteness HP Automixx 35%	12	5,60	0,149	0,022	5,47	5,96
Dash 30%	12	6,26	0,300	0,090	5,82	6,70

En la Tabla 5 se comparan los diferentes pares formados entre los tiempos de aplicación clínica del gel aclarador de peróxido de hidrógeno marca Whiteness HP Maxx al 35%. En ella se observa que existe una diferencia significativa entre T0 con T15 (P = 0,024), con T30 (P = 0,012) y con T45 (P = 0,012); sin embargo, vemos que no existe esta diferencia entre T15 y T30 (P = 0,660). Es decir, el pH desciende significativamente desde el tiempo inicial hasta el final (45 min), pero entre los 15 y 30 minutos este descenso no es significativo.

**Tabla 5:** Comparación del pH en los diferentes tiempos de aplicación clínica del gel aclarador FGM Whiteness HP 35%

SIGNIFICANCIA ESTADÍSTICA WHITENESS HP MAXX 35%				
Tiempos			Pares	
	Media ± (DE)	P1		P2
			T0 VS T15	0,024**
T0	5,99 ± 0,133			
			T0 VS T30	0,012**
T15	5,66 ± 0,153			
		p<0,001*	T0 VS T45	0,012**
T30	5,55 ± 0,074			
			T30 VS T15	0,660**
T45	5,46 ± 0,089			
			T45 VS T15	0,030**
			T45 VS T30	0,018**

*Prueba de Friedman

**Prueba de Wilcoxon con corrección de Bonferroni

En la Tabla 6 se comparan de igual manera los diferentes pares formados ente los tiempos de aplicación clínica del gel aclarador marca Lase Peroxide Sensy al 35%. Se observa que no hay diferencia estadísticamente significativa entre T0 y T15 (P = 0,246); no obstante, a partir de los 15 minutos de aplicación, sí hay diferencia significativa entre los tiempos; T15 con T30 (P = 0,018), T15 con T45 (P = 0,012) y T30 con T45 (P = 0,012). Esto quiere decir que el pH no desciende significativamente hasta los 15 minutos, pero que a partir de ese tiempo su descenso comienza a ser significativo entre tiempo y tiempo de aplicación.

**Tabla 6:** Comparación del pH en los diferentes tiempos de aplicación clínica del gel aclarador DMC Lase Peroxide 35%

SIGNIFICANCIA ESTADÍSTICA LASE PEROXIDE 35%				
Tiempos			Pares	
	Media ± (DE)	P1		P2
			T0 VS T15	0,246**
T0	6,34 ± 0,400			
			T0 VS T30	0,048**
T15	6,00 ± 0,137			
		p<0,001*	T0 VS T45	0,024**
T30	5,74 ± 0,081			
			T30 VS T15	0,018**
T45	5,62 ± 0,078			
			T45 VS T15	0,012**
			T45 VS T30	0,012**

*Prueba de Friedman

**Prueba de Wilcoxon con corrección de Bonferroni

En la Tabla 7 se comparan los pares formados entre los tiempos de aplicación clínica del gel aclarador marca Whiteness HP Automixx al 35%. Se observa que sí existen diferencias estadísticamente significativas entre el tiempo inicial de aplicación y el resto de los tiempos: T0 con T15 (P = 0,012), T0 con T30 (P = 0,012), T0 con T45 (P = 0,012), T15 con T30 (P = 0,012), T15 con T45 (P = 0,012); sin embargo, no sucede lo mismo entre los 30 y 45 minutos de aplicación T30 con T45 (P = 0,138). Esto significa que no hay un descenso significativo del pH entre los 30 y 45 minutos.

**Tabla 7:** Comparación de significancia estadística de la variación del pH en los diferentes tiempos de aplicación clínica del gel aclarador FGM Whiteness HP Automixx 35%

SIGNIFICANCIA ESTADÍSTICA WHITENESS HP AUTOMIXX 35%				
Tiempos			Pares	
	Media ± (DE)	P1		P2
			T0 VS T15	0,012**
T0	6,65 ± 0,493			
			T0 VS T30	0,012**
T15	6,00 ± 0,327			
		p<0,001*	T0 VS T45	0,012*
T30	5,67 ± 0,198			
			T30 VS T15	0,012**
T45	5,60 ± 0,149			
			T45 VS T15	0,012**
			T45 VS T30	0,138**

*Prueba de Friedman

**Prueba de Wilcoxon con corrección de Bonferroni

En la Tabla 8 se comparan los pares formados entre los tiempos de aplicación clínica del gel aclarador marca Dash al 30%. Observamos que existe únicamente una diferencia significativa estadísticamente entre el tiempo inicial de aplicación y el tiempo final T0 con T45 (P = 0,006), los demás tiempos no muestran mayores diferencias entre sí, T0 con T15 (P = 0,519), T0 con T30 (P = 0,212), T15 con T30 (P = 1,000), T15 con T45 (P = 0,750) y T30 con T45 (P = 1,000). Es decir, en este caso, el pH ha aumentado significativamente desde el tiempo inicial comparándolo con el tiempo final de aplicación.

**Tabla 8:** Comparación del pH en los diferentes tiempos de aplicación clínica del gel aclarador Philips Dash 30%

SIGNIFICANCIA ESTADÍSTICA DASH 30%				
Tiempos			Pares	
	Media ± (DE)	P1		P2
			T0 VS T15	0,519**
T0	5,71 ± 0,404			
			T0 VS T30	0,212**
T15	6,00 ± 0,532			
		0,016(p>0.001)	T0 VS T45	0,006**
T30	6,07 ± 0,366			
			T30 VS T15	1,00**
T45	6,26 ± 0,300			
			T45 VS T15	0,750**
			T45 VS T30	1,00**

*Prueba de Anova

**Prueba de Bonferroni

## DISCUSIÓN

El propósito de este estudio fue evaluar la estabilidad del pH de cuatro geles aclaradores a base de peróxido de hidrógeno (Whiteness HP Maxx 35% FGM, Lase Peroxide Sensy 35% DMC, Whiteness HP Automixx 35% FGM y Dash 30% Philips) en cuatro intervalos de tiempo: 0’, 15’, 30’ y 45’.

Se evidenció una disminución del pH, con diferencia significativa entre algunos intervalos de tiempo de los geles Whiteness HP Maxx 35%, Lase Peroxide Sensy 35% y Whiteness HP Automixx 35%, por lo tanto, la hipótesis nula fue rechazada. En el caso del gel aclarador Dash 30% se evidenció, por el contrario, un aumento del pH que fue significativo solo entre el tiempo inicial (T0) y el final (T45).

Muchos de los productos aclaradores presentan valores bajos de pH para asegurar la estabilidad del peróxido de hidrógeno (1). Como ya se dijo, se observó que hubo una tendencia a la disminución del pH. Estos resultados son similares a los del estudio de Price et al. (2000), que demostraron que los geles aclaradores a base de peróxido de hidrógeno pueden mostrar valores bajos de pH e ir disminuyendo progresivamente a través del tiempo. 

Diversos estudios han evaluado el pH de distintos geles aclaradores ^10^^,^^12^^,^^14^^,^^15^ ya que este es considerado un factor muy importante por ser el que determina el rango de acción del proceso aclarador; mientras mayor sea el pH, se producirán más radicales libres ^10^. Por otro lado, el nivel de pH de un agente aclarador se relaciona directamente con las probables alteraciones micromorfológicas que sufriría el esmalte durante un aclaramiento dental (8). Se sabe que el pH crítico para el esmalte es de 5,5 ^13^, y muchos de los geles aclaradores que existen muestran que están por debajo de ese rango ^14^. La presente investigación evidenció que los cuatro agentes evaluados no tenían un valor menor a 5,5 durante los cuatro intervalos de tiempo considerados, pero sí mostraron valores muy cercanos, por lo que es válido mencionar que la preservación del producto es igual de importante que la preservación de la estructura dentaria para los fabricantes. 

Sun et al. (2011) mostraron que tanto los productos con pH ácido como con pH neutro, tuvieron la misma eficacia en el aclaramiento; sin embargo, los geles con pH ácido causaron un 30% más de efectos adversos en la superficie del esmalte dental. Ese hallazgo indica que la desmineralización de la superficie del esmalte parece estar causada principalmente por el pH bajo en lugar de que sea por el peróxido de hidrógeno en sí ^39^.

Los promedios del pH por intervalos de tiempo y por marcas encontrados en este estudio, si bien no son críticos para el esmalte y además fueron tomados de dientes bovinos como muestra, al estar cerca de ese nivel crítico invita a tomar en cuenta que en la parte clínica ese aspecto podría relacionarse con la sensibilidad que existe durante un aclaramiento. Arana (2013) menciona que la desmineralización de la superficie del esmalte causada por el pH bajo de los agentes aclaradores podría tener un efecto sobre la actividad biodinámica de los mismos, lo que aumenta los procesos erosivos; clínicamente, el aumento de la porosidad permite que el agente aclarador penetre más fácilmente a través del esmalte y la dentina hacia la pulpa produciendo una injuria a nivel pulpar que se traduciría en la sensibilidad por aclaramiento dental ^13^.

Ha sido reportado por Price et al. (2000) que mientras más elevada sea la concentración del peróxido de hidrógeno de un gel, más ácido será el pH ^14^. Como se recuerda, la concentración del gel aclarador Dash es del 30%, menor a las concentraciones del resto de marcas (35%), y fue el único grupo que tuvo un incremento de los valores del pH a través del tiempo de aplicación clínica; esto puede deberse a que, dentro de su composición, además de una menor concentración de peróxido de hidrógeno, presenta hidróxido de amonio (NH4OH), el cual es una solución de amoniaco en agua que tiene propiedades alcalinas y se usa como regulador del pH. Price et al., en su estudio, también afirman que la liberación de amonio (o hidroxilo de amonio) eleva el pH del gel aclarador a los 15 minutos, con lo que es capaz de alcalinizarlo ^14^. Esto coincide con los resultados del presente estudio, que muestra que el grupo Dash 30% a los 15 minutos ya mostraba valores más elevados que al inicio de la aplicación.

Otro estudio que presentó resultados similares fue el de Trentino et al. (2015), quienes evaluaron el pH de 7 marcas de geles aclaradores a base de peróxido de hidrógeno con diferentes concentraciones: Whiteness HP Maxx 35%, Lase Peroxide 35%, Lase Peroxide II 25%, Lase Peroxide Lite 15%, Whitegold Office 35%, Whiteness HP Blue Calcium 35% y Whiteness HP Blue Calcium 20%. Utilizaron un pHmetro digital Sentron y registraron los valores de pH de cada muestra a los 30 segundos de aplicación en la superficie del diente bovino (tiempo inicial) y 30 segundos después de que se cumpla el tiempo de aplicación del gel (tiempo final). Los resultados mostraron que los valores de pH de los geles aclaradores tendieron a disminuir a través del tiempo inicial al final, con la excepción de las marcas Whitegold Office 35% y Whiteness HP Blue Calcium 35%, las cuales mostraron valores más altos de pH en el tiempo final ^10^. 

Actualmente, la mayoría de los agentes aclaradores a base de peróxido de hidrógeno aún presentan valores ácidos de pH con el fin de que dicho componente se mantenga estable y no se descomponga muy rápidamente ^(2, 3)^, y además que su composición también consta de ingredientes como glicerina, sales, saborizantes, entre otros, los cuales podrían alterar o manipular sus pH a través del tiempo desde su activación. Por tanto, es importante continuar estudiando los efectos que estos componentes pueden tener no solo en el comportamiento del peróxido de hidrógeno, sino también en el pH del gel aclarador, ya que finalmente estos cambios podrían afectar al tejido dentario en un determinado tiempo de aplicación clínica.

Por otro lado, es importante mencionar también que, al realizar un aclaramiento dental, la temperatura intraoral puede afectar los niveles de pH de agentes aclaradores durante el procedimiento clínico, por lo que es necesario realizar investigaciones in vivo sobre este tema.

Se sugiere, en próximos estudios, evaluar el pH de un mayor número de geles aclaradores y, en lo posible, realizarlo en pacientes, según las instrucciones del fabricante en cuanto a manipulación y tiempos de exposición con el fin de determinar el comportamiento del pH en el medio oral y las variaciones que puede tener.

**Figura 1:**
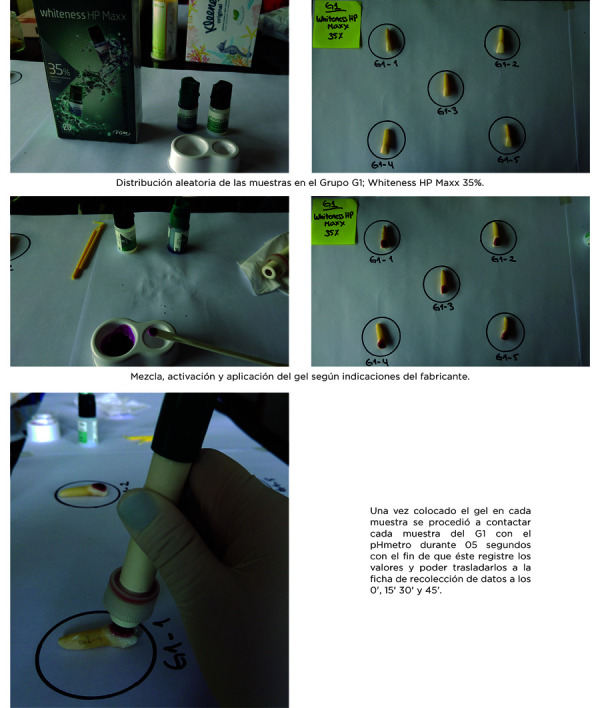
Secuencia de pasos por grupo de trabajo

## CONCLUSIONES

• Existió una tendencia a la disminución del pH de los geles aclaradores a través del tiempo de aplicación clínica en todas las marcas estudiadas, con excepción de la marca Dash 30%, la cual mostró un aumento.
• El pH de la marca Whiteness HP Maxx 35% descendió significativamente desde el tiempo inicial hasta el final (45 min), pero entre los 15 y 30 minutos este descenso no es significativo.
• En el gel aclarador Lase Peroxide 35%, el pH no desciende significativamente hasta los 15 minutos, pero a partir de ese tiempo su descenso comienza a ser significativo entre tiempo y tiempo de aplicación.
• En la marca Whiteness HP Automixx 35% existen diferencias estadísticamente significativas entre el pH del tiempo inicial de aplicación y el resto de los tiempos; sin embargo, no sucede lo mismo entre los 30 y 45 minutos de aplicación. Esto significa que no hay un descenso significativo del pH en el lapso mencionado.
• La marca Dash 30% muestra una diferencia significativa entre el tiempo inicial de aplicación y el tiempo final (45 min), el pH aumentó desde el tiempo inicial al compararlo con el tiempo final de aplicación.

## Fuente de financiamiento:

Autofinanciado
